# Serotonin Deficiency Exacerbates Acetaminophen-Induced Liver Toxicity In Mice

**DOI:** 10.1038/srep08098

**Published:** 2015-01-29

**Authors:** Jingyao Zhang, Sidong Song, Qing Pang, Ruiyao Zhang, Lei Zhou, Sushun Liu, Fandi Meng, Qifei Wu, Chang Liu

**Affiliations:** 1 Department of Hepatobiliary Surgery, The First Affiliated Hospital of Xi'an Jiaotong University; NO.277 Yanta West Road, Xi'an Shaanxi 710061, People's Republic of China; 2 Departments of Medicine (Division of Molecular and Vascular Biology), Center for Vascular Biology Research, Beth Israel Deaconess Medical Center and Harvard Medical School. Boston. U.S.A; 3 Department of Thoracic Surgery, The First Affiliated Hospital of Xi'an Jiaotong University; NO.277 Yanta West Road, Xi'an Shaanxi 710061, People's Republic of China

## Abstract

Acetaminophen (APAP) overdose is a major cause of acute liver failure. Peripheral 5-hydroxytryptamine (serotonin, 5-HT) is a cytoprotective neurotransmitter which is also involved in the hepatic physiological and pathological process. This study seeks to investigate the mechanisms involved in APAP-induced hepatotoxicity, as well as the role of 5-HT in the liver's response to APAP toxicity. We induced APAP hepatotoxicity in mice either sufficient of serotonin (wild-type mice and *TPH1-/-* plus 5- Hydroxytryptophan (5-HTP)) or lacking peripheral serotonin (*Tph1-/-* and wild-type mice plus p-chlorophenylalanine (PCPA)).Mice with sufficient 5-HT exposed to acetaminophen have a significantly lower mortality rate and a better outcome compared with mice deficient of 5-HT. This difference is at least partially attributable to a decreased level of inflammation, oxidative stress and endoplasmic reticulum (ER) stress, Glutathione (GSH) depletion, peroxynitrite formation, hepatocyte apoptosis, elevated hepatocyte proliferation, activation of 5-HT2B receptor, less activated c-Jun NH_2_-terminal kinase (JNK) and hypoxia-inducible factor (HIF)-1α in the mice sufficient of 5-HT versus mice deficient of 5-HT. We thus propose a physiological function of serotonin that serotonin could ameliorate APAP-induced liver injury mainly through inhibiting hepatocyte apoptosis ER stress and promoting liver regeneration.

Acetaminophen (N-acetyl-p-aminophenol, APAP), a widely used analgesic and antipyretic drug, is believed to be safe within therapeutic doses. However, an accidental or an intentional APAP overdose often causes acute liver failure with high morbidity and mortality^1^,^2^. The underlying mechanism of APAP hepatotoxicity is thought to be conversion of APAP to N-acetyl-p-benzoquinone imine (NAPQI), which is mediated by members of the cytochrome P450 family, especially CYP2E1. NAPQI can be immediately conjugated with glutathione (GSH) to form the non-toxic metabolites cysteine^3^,^4^. Once the intracellular pool of GSH is exhausted, NAPQI covalently combines with cellular proteins, which results in mitochondrial dysfunction and oxidative stress^5^. This type of covalent binding inactivates important functional proteins, leading to hepatic cell death and enzymes like alanine aminotransferase (ALT) release into plasma^6^. Both the intracellular (mitochondria) and extracellular (inflammatory cells) reactive oxygen species (ROS) contribute to the liver injury^7^,^8^. Oxidative stress induces c-Jun NH_2_-terminal kinase (JNK) activation, which would further promote mitochondrial dysfunction and mitochondrial permeability transition, amplify oxidative stress and lead to sustained JNK activation, and ultimately cause cell death. Previous studies revealed that JNK activation plays a major role in APAP-induced hepatotoxicity which can be inhibited by genetic depletion or pharmaceutic inhibitor^9^,^10^,^11^.

Serotonin, as well-known as 5-hydroxytryptamine (5-HT), a small monoamine molecule primarily known as a neurotransmitter, is involved in the regulation of various physiologic procedures including cognition, mood, aggression, mating, feeding, and sleep^12^. Peripherally, serotonin mediates vascular contraction and relaxation, cell proliferation, apoptosis, and platelet aggregation. To date, 15 different 5-HT receptor subtypes have been identified^13^. Except for its major role in central nervous system, recent studies show that 5-HT plays a crucial role in liver physiology and pathology. To be specific, serotonin is a double-edged sword which is both a friend and a foe to the liver^14^. On the one hand, serotonin expedites liver regeneration after partial hepatectomy in various rodent models and promotes tissue repair after ischemia/reperfusion injury^15^,^16^. It is also beneficial to the stabilization of hepatic microcirculation and prevents small-for-size liver graft failure^17^. Similarly, serotonin receptor agonists improve the sinusoidal perfusion of aged liver and restore the deficient liver regeneration in old mice which shows an effect of age defying^18^. Besides, serotonin can also regulate regeneration of the biliary tree by targeting cholangiocytes through an autocrine/paracrine signal way^19^. But, on the other hand, studies reveal that serotonin contribute to non-alcoholic fatty liver diseases (NAFLD) in rodent models through its degradation products, the reactive oxygen species^20^. Serotonin aggravates viral hepatitis, and plays a crucial role in the progression of hepatic fibrosis^21^,^22^. Serotonin may also facilitate tumor growth of primary liver carcinoma like cholangiocarcinoma and hepatocellular carcinoma^23^,^24^. So far the role of 5-HT in the APAP-induced hepatotoxicity is still unknown. In this study, we focused on the effect of 5-HT on the APAP-induced hepatotoxicity and the relevant mechanisms in mice models.

## Methods

### Experimental animals

The study was conducted using male *TPH1* knockout mice and wild-type C57BL/6 mice (4–6 weeks, 21–26 g) (Animal Feeding Center of Xi'an Jiaotong University Health Science Center). The *TPH1*−/− mice were characterized elsewhere^16^,^17^,^25^. All mice were housed (5 per cage) in clear, pathogen-free polycarbonate cages in the animal care facility, and were fed with a standard animal diet and water *ad libitum* under controlled temperature conditions with 12 h light-dark cycles. All animal experiments were performed in accordance with the guidelines of China Council on Animal Care and Use. All animal procedures carried out in this study were reviewed, approved, and supervised by the Institutional Animal Care and Use Committee of the Ethics Committee of Xi'an Jiaotong University Health Science Center, China. All animals were kept and the experiments were performed in accordance with the European Community guidelines for the use of experimental animals (86/609/EEC).

### Study design

Mice used in the study were randomly allocated into the following groups: (1) Normal control group: wild type mice and *TPH1* knockout mice were treated with 0.9% saline solution through intraperitoneal injection (because of the same results, they served as control group together). (2) WT group: wild type mice were treated with APAP (suspended in saline solution, in a final volume of 0.25 mL) through intraperitoneal injection (i.p). (3) WT+PCPCA group: wild type mice were treated with APAP and p-chlorophenylalanine (PCPA, TPH1-inhibitor, at a dose of 150 mg/kg/day for 3 days by subcutaneous injection before APAP treatment) i.p. as previously reported^25^. (4)KO group: *TPH1* knockout mice were treated i.p with APAP (TPH1, the rate-limiting enzyme for the synthesis of peripheral serotonin). (5)KO+5-HTP group: *TPH1*knockout mice were treated i.p with APAP and 5- Hydroxytryptophan (5-HTP, precursor of 5-HT which can reload the 5-HT content, at a dose of 75 mg/kg/day for 3 days by subcutaneous injection before APAP administration) as previously reported^19^. In another set of experiments, animals (n = 15 for each group) were randomly divided as described above and monitored for assessing mortality for 5 days after a lethal dose of APAP (600 mg/kg) or saline administration.

### Measurement of liver function

Serum ALT, AST and alkaline phosphate activities, as well as bilirubin levels were determined by using an Olympus AU5400 Automatic Biochemical Analyzer (Olympus, Tokyo, Japan) in the Department of Inspection, The First Affiliated Hospital of Xi'an Jiaotong University^26^.

### Cytokine measurement in murine serum

Levels of serum 5-HT, TNF-α and IL-6 were measured with a commercial ELISA kit following the instructions of the manufacturer (Dakewe, Shenzhen, China).

### Liver enzymatic activity assay

The liver tissue was homogenized, and tissue myeloperoxidase (MPO), malonaldehyde (MDA), GSH, glutathione peroxidase (GSH-px) activities were measured using the activity assay kits from NanJing JianCheng Bioengineering Institute according to the manufacturer's instructions.

### Histological study

Liver samples were excised and fixed in 10% formalin solution and embedded in paraffin after completion of the routine follow-up. Serial sections of 5-μm thickness were obtained and stained with Hematoxylin&Eosin (H&E) to evaluate morphology. The results were examined in a blind fashion by two researchers. For electron microscopy examination, liver tissues were prefixed after harvesting immediately with 1.5% glutaraldehyde and 0.8% paraformaldehyde (0.1 mol/L cacodylate buffer) at room temperature and postfixed in an aqueous solution of 1% OsO_4_ and 1.5% K_4_(FeCN)_6_. Then the specimens were embedded into Epon by routine procedures. Ultrathin sections (50 nm) were contrasted with lead citrate and uranyl acetate and studied with a CM100 Transmission Electron Microscope.

### Transferase-mediated dUTP nick end-labeling (TUNEL) assay

Apoptotic cells were detected by the TUNEL method using an in situ cell detection kit for the detection and quantification of apoptosis at a single-cell level. Staining of tissue sections was performed according to the manufacturer's protocol. Paraffin-embedded sections were dewaxed in xylene and rehydrated by passing through a graded series of ethanol solutions, ending with phosphate-buffered saline. Sections were permeabilized with proteinase K (20 μg/mL in 10 mmol/L Tris-HCl, pH 7.4–8.0) at 37°C for 15 min. After washing, sections were stained with fluorescent anti-TdT. Sections were viewed and photographed using standard fluorescent microscopic techniques.

### Immunohistochemistry (IHC)

IHC analysis was performed with 4-hydroxynonenal (4-HNE), Nitrotyrosine, TUNEL apoptotic staining, BrdU, Ki-67, PCNA antibodies and HIF-1α, using methods as previously described^27^. Mice given BrdUrd (0.5 mg/ml) in drinking water for 4 days were analyzed immunohistochemically for liver nuclear-labeling indices.

### RNA isolation and quantitative reverse transcription–polymerase chain reaction (qRT–PCR) analysis

Liver tissue samples from each group were snap-frozen in liquid nitrogen and stored at −70°C until use. Total RNA was isolated from cells using the RNAfast200 Kit (Fastagen Biotech, Shanghai, China). Reverse transcription was performed using the PrimeScript RT reagent Kit (TaKaRa Biotechnology, Dalian, China). The mRNA expression was assayed in triplicate and normalized to the β-actin mRNA expression. The relative levels were calculated using the Comparative-Ct Method (ΔΔCt method). The primers used in the study were listed in Supplementary Table 1.

### Western blotting

The monoclonal antibodies used in this research were purchased from Neomarker, Santacruz, Kangcheng for Sigma and Cell signaling. Protein concentration was determined by BCA method. Western blotting was performed as previously described^28^.

### Statistical analysis

The survival and mortality rates are expressed as percentages. The measurement data are expressed as mean ± standard deviation (SD). Differences between experimental and control groups were assessed by either t test or the analysis of variance (ANOVA), as applicable, using SPSS 18.0 (SPSS, 165 Inc.). A p-value of less than 0.05 was considered statistically significant.

## Results

### TPH1-/- mice are more susceptible to acetaminophen-induced hepatotoxicity

**Figure 1:**
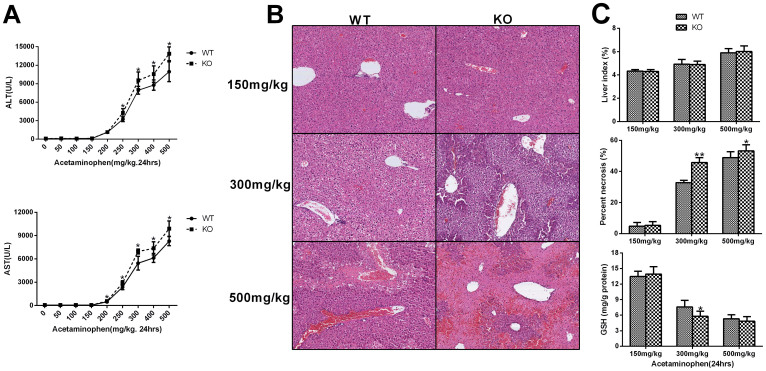
Dose-response for the effect of APAP on serum ALT and AST, histological examination, liver index and hepatic GSH in Wild-type and *TPH1-/-* mice. Mice (n = 6) were given an i.p. injection of acetaminophen (50–400 mg/kg for 24 hours). The liver injury in surviving mice was measured by (A) serum ALT and AST, and Liver tissue were obtained at 24 hours after APAP administration at the dose of 150, 300 and 500 mg/kg. (B) H&E staining, (C) liver index, hepatic necrosis area, and hepatic GSH content were adopted to evaluate the different hepatotoxicity between Wild-type and *TPH1-/-* mice. The data are presented as mean ± S.D. *P < .0.05 vs WT group, **P < .0.01 vs WT group.

**Figure 2:**
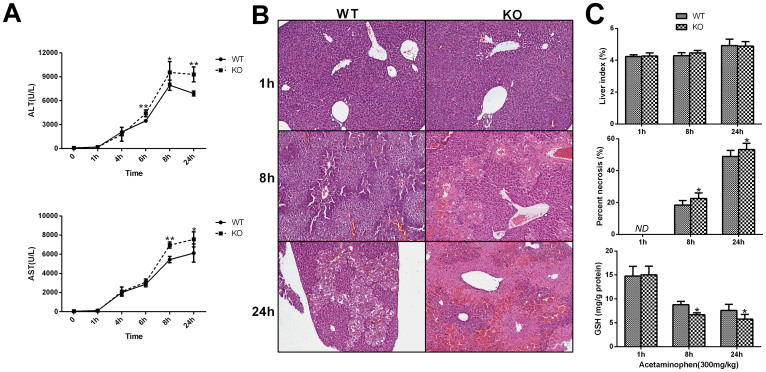
Time course for the effect of APAP on serum ALT and AST, histological examination, liver index and hepatic GSH in Wild-type and *TPH1-/-* mice. Mice (n = 6) were treated with a 300 mg/kg dose of APAP and sacrificed at the indicated times, and serum and liver were collected. The 0 time is the mice in both WT and KO receiving no APAP administration. (A) Serum ALT and AST levels. Liver tissue were obtained at 1, 8, and 24 hours after a dose of 300 mg/kg APAP administration. (B) H&E staining, (C) liver index, hepatic necrosis area and hepatic GSH levels in liver were used to assess the difference between Wild-type and *TPH1-/-* mice. The data are presented as mean ± S.D. *P < .0.05 vs WT group, **P < .0.01 vs WT group.

Acetaminophen produced a dose-dependent hepatotoxicity. Liver injury was aggravated as the acetaminophen dose increases. *TPH1* null mice were more susceptible than wild type mice to acetaminophen-induced hepatotoxicity at the doses of 250–500 mg/kg, as evidenced by marked increases in serum activities of ALT and AST (Figure 1A). In parallel with serum enzyme activities, more severe necrosis was observed in *TPH1* null mice than in wild type mice after acetaminophen administration (Figure 1B). Contemporarily, the depletion of GSH was more rapid in *TPH1* knockout mice. (Figure 1C). Then we studied the time course for the effect of APAP on hepatotoxicity in WT and KO mice. Mice were administered 300 mg/kg APAP and sacrificed at 0, 1, 4, 6, 8 and 24 hours after APAP administration. The development of hepatotoxicity was evaluated with the serum levels of ALT and AST and the histopathological evaluation. The time course of the APAP-induced increases in serum ALT and AST is shown in Figure 2. In WT and KO mice, ALT and AST levels were significantly increased by 4 hours. By comparison, the serum ALT levels in the WT mice paralleled those observed in the KO mice but seemed delayed. The serum ALT levels at 6, 8 and 24 hours in the WT mice were significantly lower than those in the KO mice. AST levels in KO mice were significantly higher than those in WT mice at 8 and 24 hours (Figure 2A). In parallel with serum enzyme activities, differences of time-dependent necrosis and hepatic GSH content were also observed in KO and WT mice (Figure 2B and 2C).

### Serotonin depletion aggravates APAP-induced liver injury in wild type mice and serotonin reloading mitigates hepatotoxicity in TPH1-/- mice

**Figure 3:**
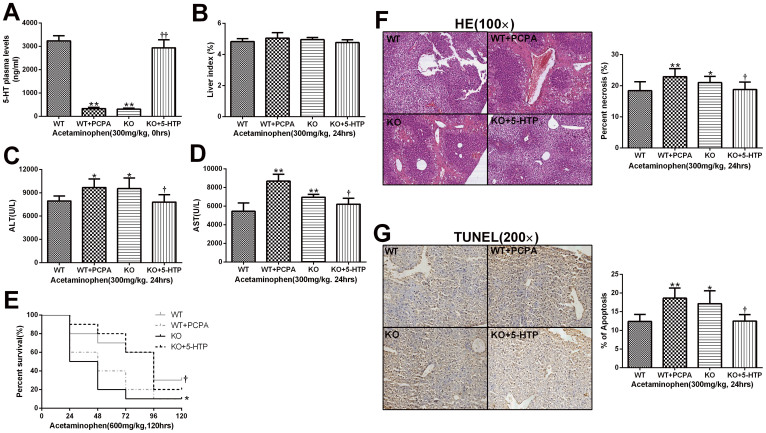
Depletion of 5-HT in wild type mice by inhibition of TPH1 aggravating APAP hepatotoxicity while reloading 5-HT in TPH1-/- mice by 5-HTP alleviating liver injury. Four groups including sufficient 5-HT (WT and KO+5-HTP groups) and deficient 5-HT (WT+PCPA and KO groups) are recruited to study the role of 5-HT in APAP hepatotoxicity. Mice (n = 6) were given an i.p. injection of acetaminophen (300 mg/kg for 24 hours), and serum and liver tissues were collected. (A), the concentration of 5-HT in the four groups. (B), Liver index of the mice after APAP administration from the four groups. (C, D), ALT and AST levels in serum. (E), Kaplan-Meier survival curve for mice (n = 15) in the four groups during 5 days after a single lethal dose of APAP (600 mg/kg). (F), the HE staining of liver sections from four groups and relative percent of necrosis area. (G), the TUNEL staining of hepatocyte apoptosis and corresponding percentage of positive cells. *P < .0.05 vs WT group, **P < .0.01 vs WT group, ^†^*P* < .0.05 vs *TPH1* knockout group, ^††^*P* < .0.01 vs *TPH1* knockout group.

To further validate the role of 5-HT in APAP-induced liver injury, wild type mice were treated with the PCPA before APAP challenge, which has been shown to be a powerful TPH1 enzyme inhibitor^25^,^29^. As demonstrated in Figure 3, PCPA significantly decreased plasma 5-HT levels in wild type mice, which was accompanied by elevations in AST, ALT levels and liver necrosis, and the reduction of 5-day's survival. Together, these findings show that genetic and pharmacologic inhibition of TPH1 significantly aggravates APAP-induced liver injury. Meanwhile, to substantiate the protective role of serotonin in experimental APAP-induced liver injury, we reloaded *Tph1-/-* mice with serotonin by injecting its precursor 5-HTP 3 days beforehand. Representative markers of liver injury (AST and ALT) reduced in the serotonin-supplemented group at 24 hours after APAP administration when compared with the KO groups (Figure 3A, 3B, 3C, 3D, 3E and 3F). Taken together, serotonin can also decrease the necrosis region of the liver and improve the 5-day's survival.

### Serotonin decreases cytokines transcription, oxidative stress and APAP-adducts formation in the liver

**Figure 4:**
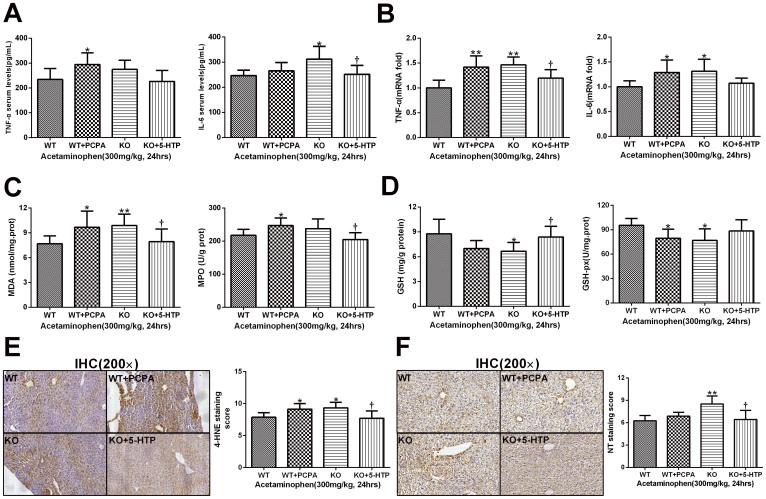
5-HT mitigates inflammation, oxidative stress and nitrotyrosine formation in the liver after APAP administration. At 24 hours after APAP challenge (300 mg/kg), serum and liver samples were collected from experimental groups to detect the levels of inflammation and oxidative stress. (A). Serum levels of TNF-α and IL-6, (B). The transcriptional levels of TNF-α and IL-6 in the liver tissue, (C). The MDA and MPO levels in the liver, (D). The levels of antioxidants (GSH and GSH-px), (E).The immumohistochemical staining of 4-Hydroxynonenal (4-HNE) and relative staining score, (F). The formation of peroxynitrite detected by immumohistochemical staining of nitrotyrosine protein adducts and relative staining score. n = 6, *P < .0.05 vs WT group, **P < .0.01 vs WT group, ^†^*P* < .0.05 vs *TPH1* knockout group, ^††^*P* < .0.01 vs *TPH1* knockout group.

To assess whether cytokines concentrations induced by APAP in the serum are influenced by 5-HT levels, we measured TNF-α and IL-6 concentrations in serum 24 hours following APAP administration. The result showed that serum levels of TNF-α and IL-6 in the WT and KO+5-HTP groups were significantly lower than the mice deficient of 5-HT (Figure 4A). Meanwhile, when detecting the mRNA levels of TNF-α and IL-6 in the liver tissues, we found that TNF-α and IL-6 had significantly higher transcriptional levels in the WT+PCPA and KO groups (Figure 4B). Meanwhile, the present study showed that APAP significantly enhanced oxidative stress (indicated by MPO and MDA levels and 4-HNE expression) in the WT+PCPA and KO groups (Figure 4C and 4E). Simultaneously, glutathione (GSH), not only an indicator of oxidative stress, but also an important role in detoxification of the acetaminophen metabolite NAPQI, was determined to ascertain whether there was a difference in its depletion in the APAP-treated mice. Treatment of wild mice with PCPA or *TPH1* knockout produced a significant reduction of GSH and GSH-px levels in liver after APAP administration when compared with the WT or KO+5-HTP groups (Figure 4D). It is well established that an APAP overdose induces peroxynitrite formation as indicated by the appearance of NT protein adducts in centrilobular hepatocytes. To investigate whether 5-HT can influence peroxynitrite formation, NT protein adducts were evaluated in the four groups 24 hours after APAP administration. The results showed that a more extensive NT staining of centrilobular hepatocytes was observed in the WT+PCPA and KO groups (Figure 4F).

### Serotonin inhibits APAP-challenged hepatocyte apoptosis in the liver

**Figure 5:**
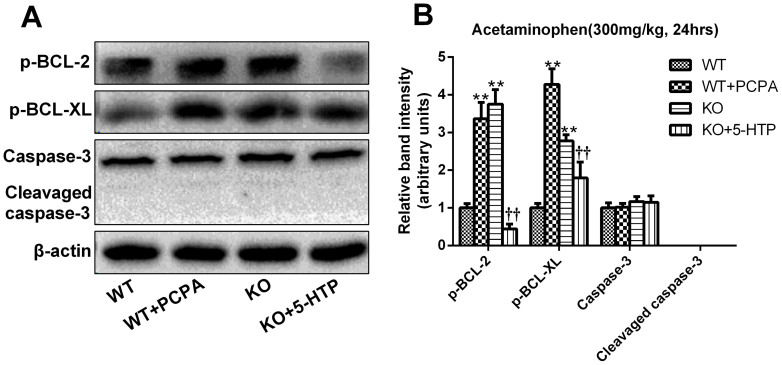
5-HT inhibits the hepatocyte apoptosis mainly by the suppression of BCL-2 proteins phosphorylation. 24 hours after APAP challenge (300 mg/kg), liver samples were collected and proteins were extracted to detect the hepatocyte apoptosis in the APAP hepatotoxicity by western blot analysis. (A, B). Bcl-2, Bcl-XL and caspase-3 protein levels. β-actin was used as an internal control. n = 6, *P < .0.05 vs WT group, **P < .0.01 vs WT group, ^†^*P* < .0.05 vs *TPH1* knockout group, ^††^*P* < .0.01 vs *TPH1* knockout group. The gels have been run under the same experimental conditions and the cropped blots images are shown in the figure and the full-length blots are presented in Supplementary Figure S1.

Acetaminophen toxicity is often associated with hepatic centrilobular necrosis while hepatocyte apoptosis is a controversial existence. To further analyze whether apoptosis would coexist with necrosis in APAP-induced hepatotoxicity, we detected APAP-induced apoptosis by TUNEL staining and investigated the possible role of Bcl-2 family proteins and caspase-3 under this pathophysiological condition. TUNEL staining showed that liver sections from mice in WT+PCPA and KO groups were more positively stained than mice in the WT and KO+5-HTP groups (Figure 3G). Contemporarily, we carried out western blot analysis of phosphorylation Bcl-2 proteins (Bcl-2 and Bcl-xL) and caspase-3 and found an apparent up-regulation in p-Bcl-2 and p-Bcl-xL expression in APAP-exposed mice. However, mice sufficient with 5-HT effectively show reduced levels of those. On the other hand, APAP-exposure increases neither caspase-3 nor cleaved-caspase-3 expression which was consistent with previous research that caspase-3 might not play an effect in the APAP-induced liver injury^30^ (Figure 5A and 5B).

### Serotonin promoted endoplasmic reticulum (ER) and inhibited ER stress after APAP challenge

**Figure 6:**
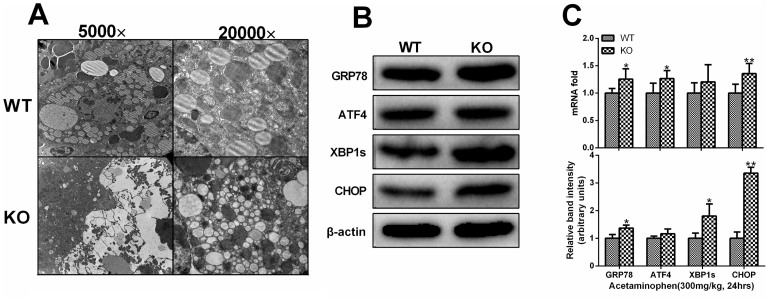
5-HT protects the organelles and inhibition of the endoplasmic reticulum (ER) stress after APAP challenge. 24 hours after APAP challenge (300 mg/kg), liver samples were collected to evaluate the ER stress. (A). Representative electron microscopy of ER tumidness, hyperplasia and dissolved, and hepatocyte megamitochondria in the liver tissue after APAP administration. (B). The ER stress associated protein levels showed by western Blot analysis. (C). The transcription and expression levels of ER stress associated gene levels. β-actin was used as an internal control. n = 6, *P < .0.05 vs WT group, **P < .0.01 vs WT group, ^†^*P* < .0.05 vs *TPH1* knockout group, ^††^*P* < .0.01 vs *TPH1* knockout group. The gels have been run under the same experimental conditions and the cropped blots images are shown in the figure and the full-length blots are presented in Supplementary Figure S1.

The endoplasmic reticulum (ER) is an important intracellular organelle that carries out multiple physiological functions. Previous studies showed that ER stress was induced during the APAP hepatotoxicity^31^. So, we detected the ER stress in the current study to determine whether ER stress can be affected by 5-HT. 24 hours after APAP administration (300 mg/kg), mice were sacrificed and the liver tissue were obtained to assess the ER stress and mitochondria injury. Subsequent analysis by electron microscopy revealed APAP induced ER tumidness, hyperplasia and dissolvent. It could also induce megamitochondria, mitochondria pyknosis, tumidness and flocculent degeneration. These injuries could be alleviated in the wild-type mice when compared with *TPH1-/-* mice (Figure 6A). Meanwhile, RT-PCR and western bolt analysis revealed that the ER stress related genes such as GRP78, ATF4, CHOP, XBP1 were more highly expressed in the mice e lack of 5-HT (Figure 6B and 6C).

### Serotonin promoted hepatocyte proliferation in vivo after APAP challenge

**Figure 7:**
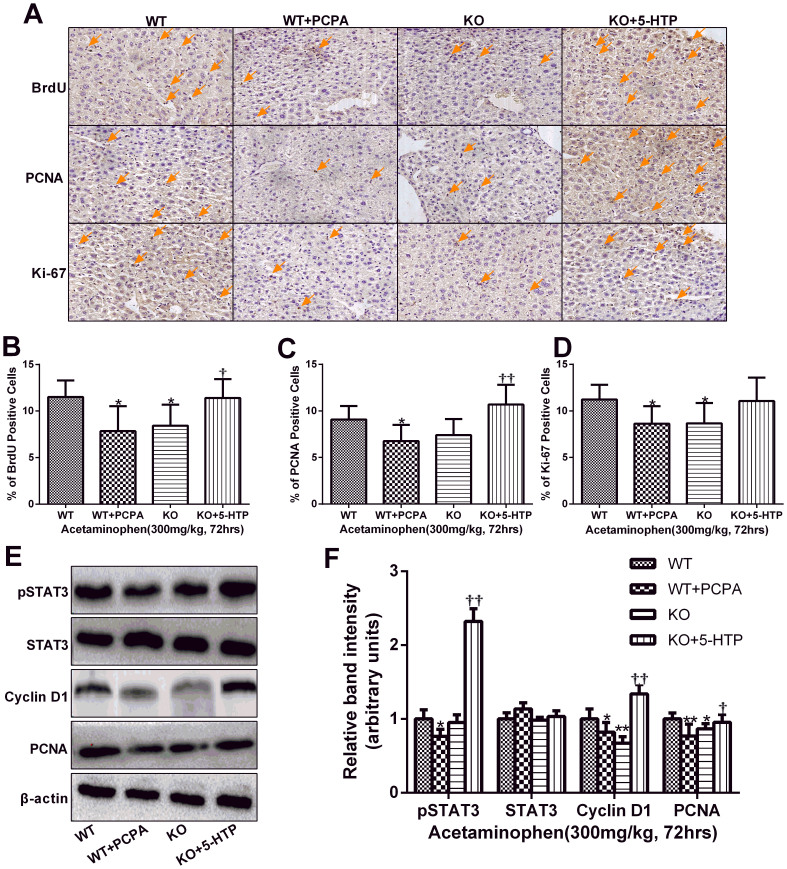
5-HT facilitates hepatocyte proliferation after APAP challenge. 72 hours after APAP administration (300 mg/kg), liver samples were collected to evaluate the effect of 5-HT on the liver regeneration. (A). Representative immunohistochemistry staining of BrdU, Ki67 and PCNA in the liver. (B–D). The quantization of hepatocyte proliferation was launched by calculating the positive cells in a microscopic vision. (E, F) The protein levels of STAT3, Cyclin D1 and PCNA, which were associated with hepatocyte proliferation, were showed by western blot analysis. β-actin was used as an internal control. n = 6, *P < .0.05 vs WT group, **P < .0.01 vs WT group, ^†^*P* < .0.05 vs *TPH1* knockout group, ^††^*P* < .0.01 vs *TPH1* knockout group. The gels have been run under the same experimental conditions and the cropped blots images are shown in the figure and the full-length blots are presented in Supplementary Figure S1.

As the former study showed that serotonin could promote the liver regeneration and hepatocyte proliferation after partial hepatectomy and hepatic ischemia/reperfusion injury^15^,^16^, we hypothesized that serotonin might also increase hepatocyte proliferation after the APAP overdose. To test this hypothesis, mice were sacrificed at 72 hours after APAP administration (300 mg/kg), and livers were harvested for BrdU, Ki67 and PCNA staining. The tissue from WT+PCPA and KO groups displayed quite a little increase in the number of positive staining hepatocytes, while 5-HT sufficiency can facilitate hepatocyte proliferation (Figure 7A,7B,7C and 7D). Meanwhile, PCNA and Cyclin D1Western blot data indicated that mice lack of 5-HT experienced a lower level of liver regeneration after the APAP overdose and exhibited significantly lower cell proliferation at 72 hours after APAP administration. To investigate the molecular mechanisms underlying the improvement of liver regeneration, we examined whether 5-HT modulated liver proliferation signaling events mainly through the transcription factor STAT3, a key protein in the hepatocyte proliferation by western blot analysis^32^,^33^. Activated STAT3 was higher expressed in the mice with 5-HT when compared with mice lack of 5-HT, which hinted that 5-HT might promote liver regeneration partially by the activation of STAT3. (Figure 7E and 7F)

### Serotonin inhibited APAP-induced phosphorylation of JNK and activation of CYP2E1

**Figure 8:**
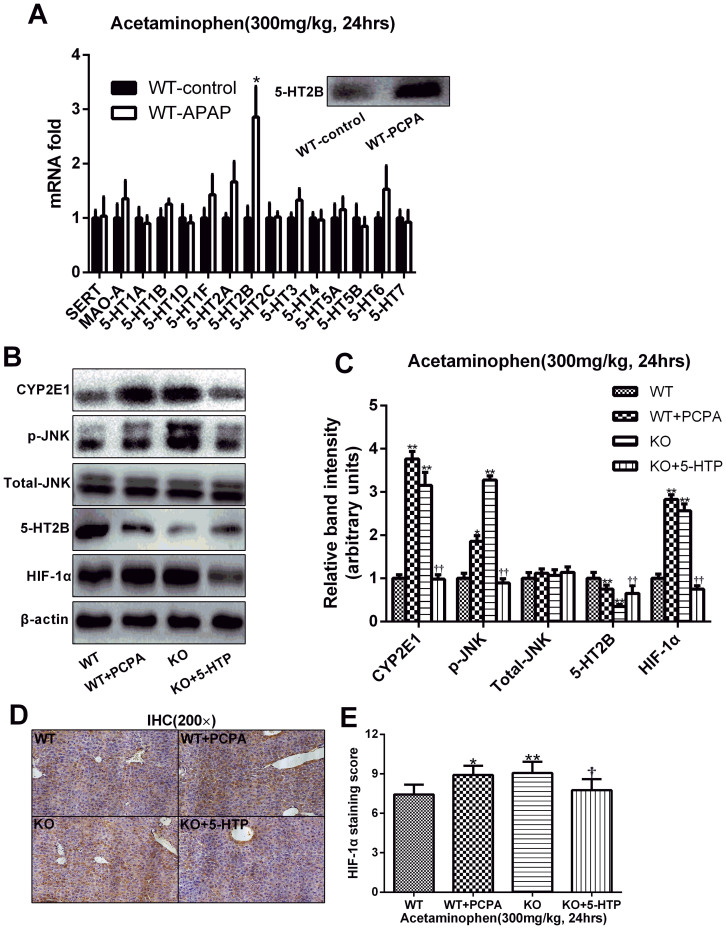
5-HT inhibits the activation of JNK to decrease the HIF-1α expression mainly by 5-HT2B receptor. To investigate the potential mechanism involved in the 5-HT protecting APAP hepatotoxicity, liver mRNAs and proteins were extracted to detect potential proteins 24 hours after APAP administration (300 mg/kg). (A) RT-PCR was performed to seek the possible 5-HT receptors, and the results showed that 5-HT2B was the target protein which was also confirmed by western blot analysis. (B, C) Western blot analysis of CYP2E1, p-JNK, Total-JNK, 5-HT2B receptor and HIF-1α. β-actin was used as an internal control. (D, E) The immumohistochemical staining of HIF-1α and relative staining score. n = 6, *P < .0.05 vs WT group, **P < .0.01 vs WT group, ^†^*P* < .0.05 vs *TPH1* knockout group, ^††^*P* < .0.01 vs *TPH1* knockout group. The gels have been run under the same experimental conditions and the cropped blots images are shown in the figure and the full-length blots are presented in Supplementary Figure S1.

5-HT receptors are the direct target of 5-HT. So we used RT-PCR to detect the specific 5-HT receptor that was involved in the APAP-induced hepatotoxicity. The result showed that 5-HT2B receptor was more expressed compared with others, which was also confirmed by the Western Blot analysis (Figure 8A). It was also very interesting that previous studies showing activation of 5-HT2B could promote hepatocyte proliferation in mice, which was in accordance with the result in our study^17^. It is known that JNK activation is mediated by oxidative stress and plays pathogenic roles in a diverse array of cellular programmes, including cell differentiation, movement, proliferation and death^34^. To investigate the mechanisms for APAP-induced necrotic hepatocytes death, we studied the JNK involved in the signal transduction in the hepatic tissues. Western blot analyses showed a significant enhancement in the expression of phospho-JNK after APAP challenge, and sufficient 5-HT could reduce its expression, whereas no significant change was found in the expression of total JNK. Furthermore, we investigated the effect of APAP on CYP2E1 protein level and found that CYP2E1 protein level was increased in the APAP-induced liver tissue while 5-HT could normalize these changes and maintain the normal physiology of the organs (Figure 8B and 8C). Meanwhile, we detected the HIF-1α, a key protein in the mechanism of APAP-induced liver injury^35^ and found that 5-HT could decrease the expression of HIF-1α by which 5-HT contributed to relieve of APAP-induced hepatotoxicity (Figure 8D and 8E).

## Discussion

Liver is the major site for drug metabolism and elimination, which makes it susceptible to drug toxicity^36^. Acetaminophen, the most commonly used analgesic drug, is believed to be safe within therapeutic doses. However, an APAP overdose always produces a centrilobular hepatic necrosis which is the main cause of acute liver failure in the western world^2^. Though decades of studies have been launched, the mechanisms of APAP-induced liver injury are not completely known. 5-HT is a small monoamine molecule primarily known for its role as a neurotransmitter. Previous studies showed that peripheral 5-HT could play an important role in the hepatic physiological and pathological process^37^,^38^. This experiment was designed to study the role of 5-HT in acetaminophen-induced hepatotoxicity. We employed mice lacking Tph1, the rate-limiting enzyme for the synthesis of peripheral serotonin, to sort as the 5-HT deficient group. We also used PCPA, an inhibitor of TPH1 as the pharmacological intervention to induce the 5-HT deficiency in the wild type mice. Meanwhile, we used 5-HTP, the precursor of 5-HT, to replenish the 5-HT deficiency in the *TPH1-/-* mice. Based on both genetic and pharmacological methods, we recruited four major groups, including two rich of 5-HT groups (WT and KO+5-HTP groups) and two deficient of 5-HT groups (WT+PCPA and KO groups), to ensure the reliability of the experimental results. Our data showed that depletion of 5-HT using genetic and pharmacologic inhibition of TPH1 resulted in an increased APAP-induced liver injury and a slower recovery. In contrast, supplementation of 5-HT with 5-HTP in *TPH1-/-* mice resulted in a delayed initiation of liver injury and an accelerated recovery after APAP treatment. Taken together, our data indicate that 5-HT can protect against APAP-induced liver injury.

After APAP administration, liver injury was detected in both KO and WT mice. The hepatotoxicity differences between the KO and WT mice were in a time and dose dependent manner. These differences were also observed in the histopathological evaluation of toxicity. Meanwhile, the depletion of glutathione and arylation of proteins by acetaminophen are important events leading to acetaminophen hepatotoxicity. In the present study, TPH1 null mice were more susceptible to acetaminophen-induced glutathione depletion compared with the WT mice. Acetaminophen-induced hepatotoxicity is thought to be mediated by a cytochrome P-450-generated intermediate, NAPQI^30^. Therefore, we determined whether TPH1 null mice had an altered metabolism of acetaminophen. Our results on hepatic CYP2E1 analysis using western blot indicate that 5-HT could influence the metabolism of APAP mainly by decreasing the hepatic CYP2E1 expression. Simultaneously, an APAP overdose induces peroxynitrite formation as indicated by the appearance of NT protein adducts in centrilobular hepatocytes^39^,^40^. To investigate whether 5-HT can affect peroxynitrite formation, NT protein adducts were evaluated by immunohistochemistry. Amore extensive NT staining of centrilobular hepatocytes was observed in the WT+PCPA and KO group when compared with the WT and KO+5-HTP mice. Oxidative damage has been proposed as a contributing mechanism of acetaminophen hepatotoxicity^41^. Oxidative stress occurs in cells when there is a disruption of cellular redox balance^42^. Acetaminophen-induced oxidative stress results in lipid peroxidation, oxidation of protein thiols, mitochondrial injury, altered calcium homeostasis, and DNA damage^43^. In the present study, TPH1 null mice were more susceptible than controls to acetaminophen-and NAPQI-induced lipid peroxidation, as evidenced by increased MDA and MPO levels, and reduced GSH and GSH-px in the liver tissues which were also important antioxidant enzymes. The immunohistochemical localization of lipid peroxidation adducts (4-HNE) at early time-points correlated with necrosis of hepatocytes around the central zone. All these data suggested that the greater susceptibility of TPH1 null mice might be partially due to the increased oxidative damage produced by acetaminophen.

The role of apoptosis in acetaminophen induced liver injury is controversial^44^,^45^. It is generally accepted that the ultimate form of hepatic damage caused by acetaminophen is necrosis^5^. But, according to the latest research, researchers tend to agree with its existence^46^,^47^. During APAP intoxication in the mouse, toxic ROS are generated which can cause apoptotic cell death including JNK activation^10^. Both in vivo and in vitro, acetaminophen can induce apoptosis by triggering the release of mitochondrial cytochrome-c and activating the apoptotic pathway^48^,^49^. Several investigators have suggested that the way of cell death induced by acetaminophen may be related to the overall acetaminophen tissue concentrations and intracellular concentrations of ATP^50^. So, in this study, we examined whether apoptosis contributes to the induction of acute liver failure caused by APAP and whether 5-HT can affect this pathological process. According to the results of the research, two anti-apoptotic proteins of the Bcl-2 family (Bcl-2 and Bcl-xL) could be phosphorylated by p-JNK. But sufficient 5-HT could decrease its expression along with JNK activation. It has also been reported that caspase inhibitors protect liver from APAP-induced injury, suggesting that apoptosis plays a vital role in initiating the events that lead to hepatic damage, but APAP does not activate execution caspases-3 and -7^51^,^52^. So we also investigated the role of caspase-3 after APAP administration and found that caspase-3 was not activated in our experiments. These data suggested that the protection of 5-HT might be partially due to the inhibition of apoptosis of hepatic cells mainly by suppression of Bcl-2 proteins phosphorylation.

Endoplasmic reticulum is an important intracellular organelle that carries out multiple physiological functions such as protein folding, posttranslational modifications, biosynthesis of fatty acids and sterols, detoxification of xenobiotics, and the storage of intracellular calcium^53^. It is also important in drug metabolism, as most phase I enzymes including cytochrome P450 oxidases and some phase II enzymes localize to the ER^54^. Hepatocytes in the liver contain abundant ER, which endows the liver with the capacity to engage in lipid and drug metabolism as well as the production of plasma proteins. ER stress occurs when the amount of protein entering the ER exceeds its folding capacity. Previous studies showed that ER stress in the liver could be induced after APAP administration which contributes to the liver injury. Uzi D et al found that inhibition of CHOP, a key protein of ER stress, could protect liver from APAP hepatotoxicity^31^. So,we firstly study the change of hepatocellular subcellular structure by electron microscope. Massive ER swelling, hyperplasia and dilation could be observed in the liver after APAP administration. Meanwhile, real-time PCR and western blot showed that the ER stress could be induced by APAP. And 5-HT could stabilize the structure of organelles and inhibit the ER stress which had the cytoprotection effect as previous studies showed.

Liver regeneration is a vital process for survival after a toxic insult. Regeneration ensures the replacement of necrotic cells and the full recovery of organ function^55^. Several studies have demonstrated that compensatory liver regeneration after APAP-induced liver injury is a critical determinant of final outcome of APAP overdose^56^,^57^,^58^. The exposure to growth factors such as hepatocyte growth factor results in the expression of cell cycle proteins. The induction of Cyclin D1 is the marker for cell cycle (G1 phase) progression in hepatocytes^33^,^59^. In current investigation, our western blot data showed that a higher level of 5-HT markedly increased the level of Cyclin D1 in the APAP challenged liver tissue. These changes in Cyclin D1 expression were associated with decreased serum ALT/AST and improved liver regeneration in wild type and TPH1-/- plus 5-HTP mice receiving APAP, suggesting that 5-HT likely facilitates activation of Cyclin D1-mediated regeneration pathways. Our data suggest that 5-HT is an important factor that promotes hepatocyte regeneration after the APAP overdose. Investigators have shown similar findings in a normothermic ischemia/reperfusion model of injury. Nocito A and others demonstrated that TPH1 knock-out mice had significantly less severe liver injury after ischemia/reperfusion, related to the accelerated hepatocyte proliferation in the knock-out mice^16^. Similarly, Lesurtel M and colleagues also demonstrated that serotonin promoted liver regeneration after partial hepatectomy which was mediated by 5-HT2A and 2B subtype serotonin receptors^15^. Meanwhile, Tian YH et al found that serotonin could protects small-for-size liver grafts from injury and prevents microcirculation and regeneration through its activation on receptor-2B^17^. These findings are similar to ours in that the 5-HT confers protection against a hepatic injury by promoting the hepatocyte proliferation and liver regeneration by the activation of 5-HT 2B receptor.

JNK is activated during oxidative stress and plays a central role in APAP-induced hepatotoxicity^60^. Hepatic JNK activation is a critical step in the intra-cellular signaling involved in APAP-induced liver injury^10^,^61^. Our data show that 5-HT depletion results in a rapid activation of JNK and that the appropriate supplementation results in a decreased activation of JNK compared with control mice. These data indicate that 5-HT may suppress JNK activation during APAP-induced liver injury. But, whether 5-HT directly affect JNK activation or induce this effect via an upstream regulator is not known. 5-HT is known to signal via multiple 5-HT receptor pathways in the liver. It is possible that 5-HT may use one of these pathways to suppress JNK signaling after APAP-induced liver injury. Meanwhile, HIF-1α was an important protein associated with JNK signaling which was also a key protein in the pathological process of APAP-induced liver injury. HIF-regulated genes include plasminogen activator inhibitor-1 (PAI-1), cell death proteins such as BNIP3 and Nix, which can all contribute to the liver injury^35^. According to the study, 5-HT may decrease the expression of HIF-1α to protect APAP hepatotoxicity.

**Figure 9:**
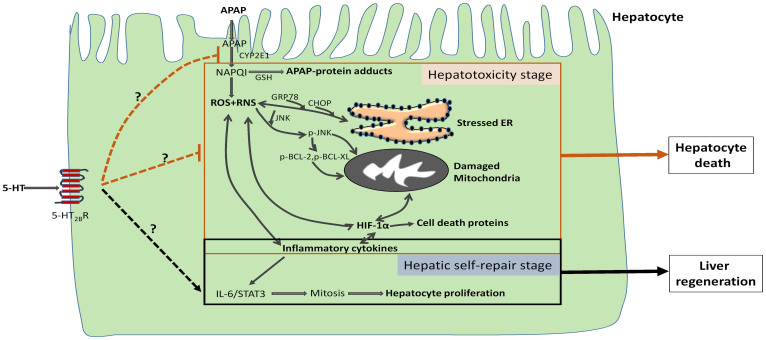
Schematic representation of the proposed role of 5-HT in APAP-induced hepatic injury. After administration into the hepatocytes, APAP is first metabolized by the CYP2E1 and generates the NAPQI, which binds with GSH and also increases ROS and RNS formation to initiate the ER stress which involved in GRP78 and CHOP activation. Increased ROS leads to the JNK phosphorylation to damage the mitochondria, which also promotes the phosphorylation of BCL-2 proteins to induce the apoptosis. Meanwhile, inflammatory factors and HIF-1α can be induced with the oxidative stress. During the hepatic self-repair stage, IL-6/STAT3 pathway can be initiated to promote the cell mitosis and facilitate the liver regeneration. In the present study, we prove that 5-HT can reduce APAP hepatotoxicity by inhibiting APAP metabolism, ROS and RNS formation and promoting the hepatocyte proliferation.

In conclusion, we describe a novel physiological role for endogenous serotonin in the protection from APAP-induced liver injury (Figure 9). Considering the pleiotropic effects of serotonin in various physiological processes, we investigated a number of potential mechanisms that might underlie the protection effect of 5-HT. This protect effect of 5-HT on APAP hepatotoxicity was at least partially attributable to a significantly decreased inflammation, oxidative stress, GSH depletion, peroxynitrite formation, hepatocyte apoptosis and ER stress, elevated hepatocyte proliferation, activation of 5-HT2B receptor and less activated JNK and HIF-1α.

## Author Contributions

J.Y.Z. designed the experiments, performed most experiments and wrote the paper; S.D.S. and Q.P. discussed the results and generated figures and supported the performance of WB; R.Y.Z. revised the paper and improved the language; L.Z. supported the performance of real time-PCR; S.S.L. contributed the revised manuscript and data analysis. F.D.M. supported the performance of WB and contributed the revised manuscript. Q.F.W. supported the performance of tissue staining; C.L. designed and directed the overall project. All authors reviewed the manuscript.

## Supplementary Material

Supplementary Information Supplementary Information
