# Supplementary material for: Serotonin Deficiency Exacerbates Acetaminophen-Induced Liver Toxicity In Mice
Source: Sci Rep. 2015 Jan 29;5:8098. doi: 10.1038/srep08098 (PMC4309973; doi:10.1038/srep08098)
Supplement: Supplementary Information [file srep08098-s1.pdf]

Supplementary Information

Title of manuscript:

Serotonin Deficiency Exacerbates Acetaminophen-Induced Liver Toxicity In in Mice

Jingyao Zhang[1], Sidong Song [1], Qing Pang[1], Ruiyao Zhang[1], Lei Zhou[1, 2], Sushun Liu[1], Fandi Meng[1], Qifei Wu[3], Chang Liu[1*]

Correspondence to: Chang Liu, MD, PHD, Department of Hepatobiliary Surgery, The First Affiliated Hospital of Xi'an Jiaotong University,

Address ： NO.277 Yanta West Road, Xi'an Shaanxi 710061, People's Republic of China.

E-mail：liuchangdoctor@gmail.com

Telephone: +86-29-85323900 **Fax**: +86-29-85324642;

1. Department of Hepatobiliary Surgery, The First Affiliated Hospital of Xi'an Jiaotong University; NO.277 Yanta West Road, Xi'an Shaanxi 710061, People's Republic of China.

2. Departments of Medicine (Division of Molecular and Vascular Biology), Center for Vascular Biology Research2, Beth Israel Deaconess Medical Center and Harvard Medical School. Boston. U.S.A.

3. .Department of Thoracic Surgery, The First Affiliated Hospital of Xi'an Jiaotong University; NO.277 Yanta West Road, Xi'an Shaanxi 710061, People's Republic of China.

Supplementary Table 1

Primer sequences used in the experiment

TNF-α	
Forward	5’-AAGCCTGTAGCCCACGTCGTA-3’
Reverse	5’-AGGTACAACCCATCGGCTGG-3’
IL-6	
Forward	5’-TCCATCCAGTTGCCTTCTTG -3’
Reverse	5’-TTCCACGATTTCCCAGAGAAC-3’
XBP1	
Forward	5’-CCTGAGCCCGGAGGAGAA-3’
Reverse	5’-CTCGAGCAGTCTGCGCTG-3’
ATF4	
Forward	5’-ACTATCTGGAGGTGGCCAAG-3’
Reverse	5’-CATCCAACGTGGTCAAGAGC-3’
ATF6	
Forward	5’-CAGTTGCTCCATCTCCTCTCC-3’
Reverse	5’-TGGGACACTGGCATTGGTTTG-3’
GRP78	
Forward	5’-CGTGGAGATCATAGCCAACG-3’
Reverse	5’-ATACGCCTCAGCAGTCTCCT-3’
CHOP	
Forward	5’-AGCCTGGTATGAGGATCTGC-3’

Reverse	5’-CTCCTGCTCCTTCTCCTTCA-3’
5-HT1A	
Forward	5’-CATCGCGCTAGACAGGTACTG-3’
Reverse	5’-CAATGAGCCAAGTGAGCGAGA-3’
5-HT1B	
Forward	5’-CGCCGACGGCTACATTTAC-3’
Reverse	5’-AGCGATTACAAAGGCGTTGGA-3’
5-HT1D	
Forward	5’-TGTCCTCTCCAATGCCTTCG-3’
Reverse	5’-TGGTGTAGGCTATGCTGATGG-3’
5-HT1F	
Forward	5’-CATTGCGGGAAACATACTGGT-3’
Reverse	5’-CCAGCAGCATATCAGCTATGG-3’
5-HT2A	
Forward	5’-CGAAGCCTCGAACTGGACAAT-3’
Reverse	5’-CCGCAATGGTGAGAATAATCACG-3’
5-HT2B	
Forward	5’-ACCTGATCCTGACTAACCGTT-3’
Reverse	5’-TGGGTATTATCACCGCGAGTAT-3’
5-HT2C	
Forward	5’-GATGGTGGACGCTTGTTTCAA-3’
Reverse	5’-GCCATGATAACGAGAATGTTGC-3’

5-HT3	
Forward	5’-CCTGGCTAACTACAAGAAGGGG-3’
Reverse	5’-TGCAGAAACTCATCAGTCCAGTA-3’
5-HT4	
Forward	5’-GATGCTAATGTGAGTTCCAACGA-3’
Reverse	5’-CAGCAGGTTGCCCAAGATG-3’
5-HT5A	
Forward	5’-ATGGATCTGCCTGTAAACTTGAC-3’
Reverse	5’-CACTCGGAAAGCTGAGAGAAAA-3’
5-HT5B	
Forward	5’-GGTGGTGCTCTTCGTCTACTG-3’
Reverse	5’-TGGAAGGTTACTGTTGCTCGG-3’
5-HT6	
Forward	5’-GCATAGCTCAGGCCGTATGTG-3’
Reverse	5’-CGCATGAAGAGGGGATAGATGA-3’
5-HT7	
Forward	5’-ATCTCGGTGTGCTTTGTCAAG-3’
Reverse	5’-GCGATGAAGACGTTGCAGAAG-3’
SERT	
Forward	5’-GACAAAGAGGACTGCCAAGAAA-3’
Reverse	5’-ATAGCCAATGACAGACAGGAGG-3’
MAO-A	

Forward	5’-CGGCAGCCAGTAGGCAGGATTT-3’
Reverse	5’-TTGAGCAGACCAGGCACGGAAG-3’
18S	
Forward	5’-AAACGGCTACCACATCCAAG-3’
Reverse	5’-CCTCCAATGGATCCTCGTTA-3’

Supplementary Figure S1.

The full-length blots in the experiment.

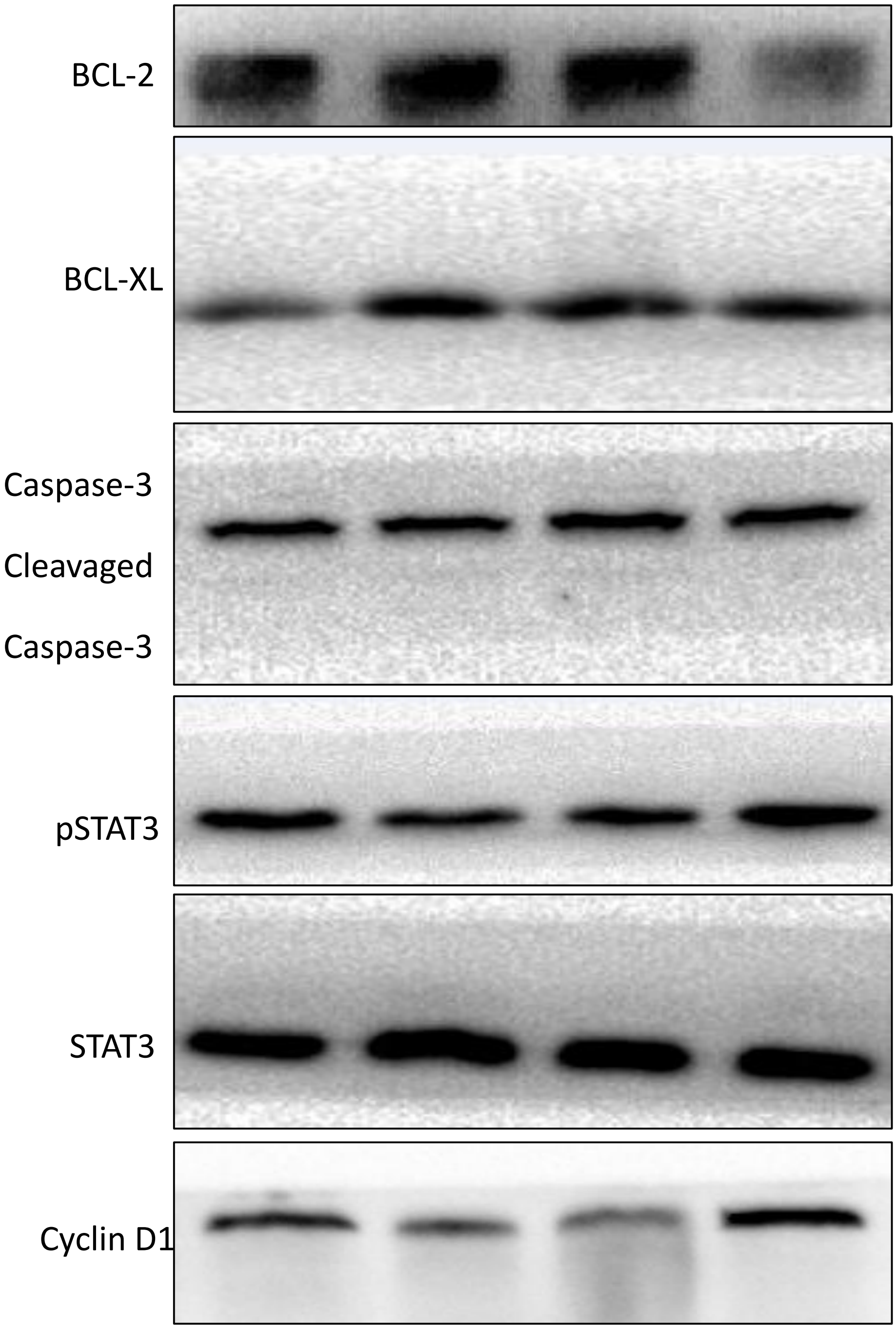

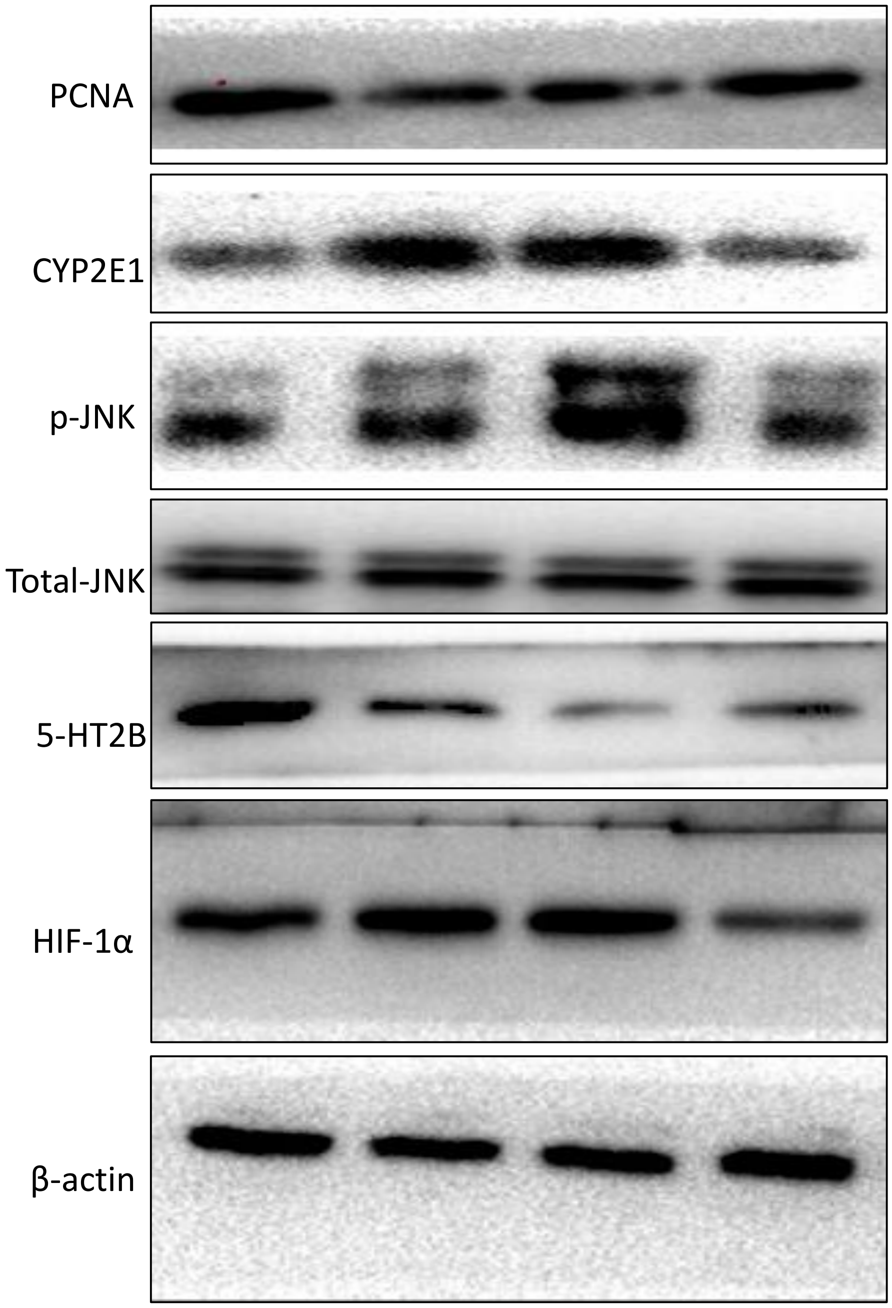
PCNA
CYP2E1
p-JNK
Total-JNK
5-HT2B
HIF-1α
β-actin

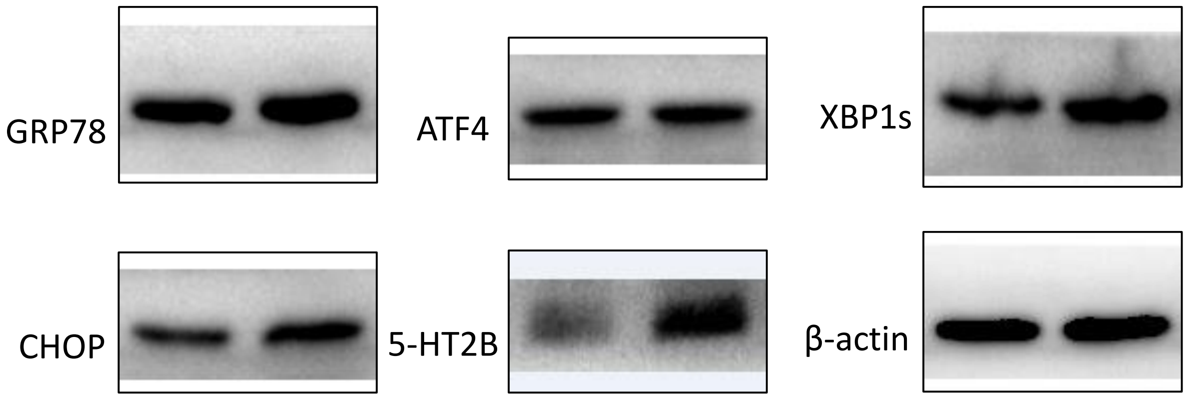
GRP78
ATF4
XBP1s
CHOP
5-HT2B
β-actin
